# Simultaneous Quantification of Antidiabetic Agents in Human Plasma by a UPLC–QToF-MS Method

**DOI:** 10.1371/journal.pone.0167107

**Published:** 2016-12-08

**Authors:** Mariana Millan Fachi, Letícia Bonancio Cerqueira, Letícia Paula Leonart, Thais Martins Guimarães de Francisco, Roberto Pontarolo

**Affiliations:** Department of Pharmacy, Federal University of Paraná, Curitiba – Paraná, Brazil; H Lee Moffitt Cancer Center and Research Institute, UNITED STATES

## Abstract

An ultra-performance liquid chromatography quadrupole time-of-flight mass spectrometry method for the simultaneous quantification of chlorpropamide, glibenclamide, gliclazide, glimepiride, metformin, nateglinide, pioglitazone, rosiglitazone, and vildagliptin in human plasma was developed and validated, using isoniazid and sulfaquinoxaline as internal standards. Following plasma protein precipitation using acetonitrile with 1% formic acid, chromatographic separation was performed on a cyano column using gradient elution with water and acetonitrile, both containing 0.1% formic acid. Detection was performed in a quadrupole time-of-flight analyzer, using electrospray ionization operated in the positive mode. Data from validation studies demonstrated that the new method is highly sensitive, selective, precise (RSD < 10%), accurate (RE < 12%), linear (r > 0.99), free of matrix and has no residual effects. The developed method was successfully applied to volunteers’ plasma samples. Hence, this method was demonstrated to be appropriate for clinical monitoring of antidiabetic agents.

## Introduction

Diabetes mellitus is characterized by hyperglycemia resulting from defects in insulin secretion, insulin action, or both [1, 2]. It is considered one of the most worrisome health problems, affecting 415 million people worldwide, which is projected to increase to 642 million people by the year 2040 [3].

In order to achieve glycemic control in type 2 diabetes (T2D), it is initially recommended that patients maintain a healthy diet and engage in regular physical activity [4]. When lifestyle modification alone is not enough to achieve glycemic targets, oral antidiabetic agents are prescribed [5].

Metformin, a drug from the biguanide class, is typically the first-line therapy used to control T2D because of its efficacy, durability, low cost, and ability to prevent weight gain and reduce risk of hypoglycemia. However, for patients with a high HbA_1c_ level (i.e., HbA_1_c ≥ 9.0) or for nonresponders to metformin after three months of treatment, the use of a second oral agent is recommended [6–10].

There are several possible combinations of antidiabetic agents; the choice of therapy is based on the individual characteristics of the patient, the pharmacological properties of the drug, and the availability of the therapy in the market, which can vary from country to country [11–13].

The choice for a second agent to be used along with metformin can be established by following the recommendations of the American Association of Clinical Endocrinologists and American College of Endocrinology [7], the American Diabetes Association and the European Association for the Study of Diabetes [8, 14], and the *Sociedade Brasileira de Diabetes* (Brazilian Society of Diabetes) [9]. It is recommended that metformin be combined with an agent of one of these therapeutic classes: sulfonylurea, thiazolidinedione, or DPP-4 inhibitors. Besides these classes, meglitinides can be used for postprandial glucose control [8].

Measurement of the plasma concentration of antidiabetic agents through a bioanalytical method is important for therapeutic monitoring and for evaluating adherence to therapy, pharmacokinetic aspects of the drug, and dosing optimization [15, 16].

Several bioanalytical methods for the quantification of antidiabetic agents in plasma have been reported in the literature; however, these methods are used for few drugs and are not suitable for the different combinations commonly used in clinical practice. In the present study, a fast and sensitive ultra-performance liquid chromatography quadrupole time of flight mass spectrometry (UPLC-QToF-MS) method was developed and validated according to the guidelines of the European Medicines Agency [17], U.S. Food and Drug Administration [18], and Brazil National Health Surveillance Agency [19]. This method was used to simultaneously quantify the levels of chlorpropamide, glibenclamide, gliclazide, glimepiride, metformin, nateglinide, pioglitazone, rosiglitazone, and vildagliptin in human plasma.

## Materials and Methods

### Reagents and Samples

High-performance liquid chromatography (HPLC) grade acetonitrile and methanol were obtained from Panreac (Barcelona, Spain). Formic acid (88%) was obtained from J.T. Baker (New Jersey, USA), and ammonium formate (97%) was obtained from Spectrum Chemical (Gardena, EUA). Ultrapure water was produced using a purification system from Millipore Corporation, USA.

**Fig 1:**
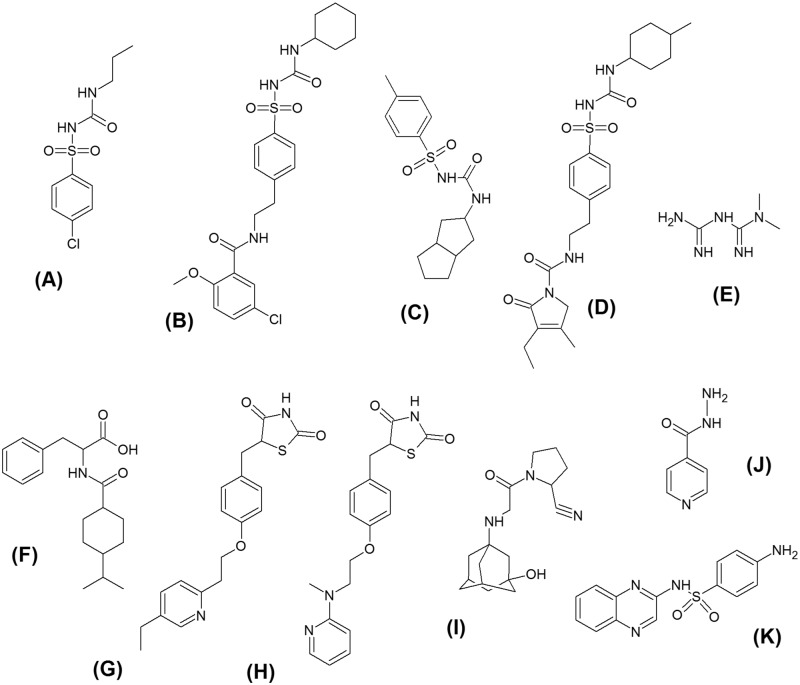
Chemical structures of (A) chlorpropamide, (B) glibenclamide, (C) gliclazide, (D) glimepiride, (E) metformin, (F) nateglinide, (G) pioglitazone, (H) rosiglitazone, (I) isoniazid, IS, and (K) sulfaquinoxaline, IS.

The metformin (99.7%), glibenclamide (99.0%), and glimepiride (99.4%) standards were purchased from United States Pharmacopoeia (Rockville, USA). Standards of chlorpropamide (99.9%), gliclazide (100.0%), and isoniazid (99.5%), which was used as the internal standard (IS), were obtained from Fiocruz/INCQS (Rio de Janeiro, Brazil). Standards of nateglinide (98.0%), pioglitazone (98.0%), rosiglitazone (98.0%), and sulfaquinoxaline (96.0%), which was used as the IS, were supplied by Sigma–Aldrich (St. Louis, USA). The vildagliptin (98.0%) standard was acquired from Ontario Chemicals (Ontario, Canada). The internal standards were selected based on their structural similarity to the analytes. The structure of each substance is shown in Fig 1.

Normal, hemolyzed, and lipemic blank plasmas were provided by the Center of Hematology of Paraná, Hemepar, Curitiba, Brazil. After centrifugation at 4,000 rpm at room temperature, the plasma was collected and stored at −40°C.

### Preparation of stock and working solutions

Stock solutions of chlorpropamide (CHL), glibenclamide (GBC), gliclazide (GCZ), glimepiride (GMP), metformin (MET), nateglinide (NAT), pioglitazone (PIO), rosiglitazone (ROS), vildagliptin (VDP), and ISs were prepared individually using acetonitrile:methanol (80:20, v/v), in order to obtain concentrations of 1 mg.mL^−1^ of each analyte. All stock solutions were stored in amber bottles at −40°C. Working standard solutions were freshly prepared every day from the stock solutions for each experiment, through appropriate dilution with acetonitrile:water (70:30, v/v), to achieve the concentrations of 1, 10 and 100 μg.mL^−1^ of analytes and ISs.

### Liquid Chromatography

Chromatography was conducted on an Acquity UPLC H-Class system (Waters Corp., Milford, USA) equipped with an autosampler (Sample Manager FTN, Waters Corp., Milford, USA) maintained at room temperature. The separation of analytes was performed on an Acquity UPLC^®^ HSS Cyano (100 x 2.1 mm, 1.8 μm) from Waters Corp. (Dublin, Ireland). The column temperature was maintained at 40°C. The analysis was performed with gradient elution using water and acetonitrile (both containing 0.1% formic acid) as mobile phases A and B, respectively. The elution order was as follows: 0–0.50 min, 2% B; 0.50–3.50 min, 2% to 70% B; 3.50–3.51 min, 70% to 95% B; 3.51–4.50, maintained at 95% B; 4.50–4.51 min, 95% to 2% B; and 4.51–6.50 min, maintained at 2%. The flow rate was 600 μL.min^-1^ and the injection volume was 5 μL.

The Waters Xevo G2-S QToF mass spectrometer (Waters Corp., Milford, USA) was connected to the UPLC system via an electrospray ionization (ESI) interface. The ESI source was performed in positive ionization mode with a capillary voltage of 0.4 kV. The temperature of the source was set at 150°C, and the desolvation temperature was set at 500°C. Nitrogen was used as the cone and desolvation gas. The cone gas flow was 50 L.h^-1^, and the desolvation gas flow was 800 L.h^-1^. All data was collected in centroid mode, acquired using MassLynx^™^ NT4.1 software, and processed using QuanLynx software (Waters Corp., Milford, USA).

### Mass Spectrometry

Mass spectrometry data was collected over a mass range of 50–600 m/z. The MS experiment was carried out under the following conditions: function 1, collision energy of 4 V. Accurate mass was determined with a 1000 ng.mL^-1^ leucine enkephalin (*m/z* 556.2771 [M+H]^+^) solution at a flow rate of 20 μL.min^-1^, which was used for lockmass. The interval mass considered in the procedure was lower than 5 ppm.

Quantification was performed using high-resolution mass data of the molecular ions [M+H]^+^ at *m/z* values of 277.0413 (chlorpropamide) (medium mass error 1.08 ppm), 494.1516 (glibenclamide) (medium mass error 0.80 ppm), 324.1381 (gliclazide) (medium mass error 1.23 ppm), 491.2328 (glimepiride) (medium mass error 1.62 ppm), 130.1092 (metformin) (medium mass error 0.76 ppm), 318.2068 (nateglinide) (medium mass error 0.62 ppm), 357.1373 (pioglitazone) (medium mass error 1.27 ppm), 358.1225 (rosiglitazone) (medium mass error 1.94 ppm), 304.2025 (vildagliptin) (medium mass error 1.31 ppm), 138.0667 (isoniazid-IS) (medium mass error 1.62 ppm), and 301.0759 (sulfaquinoxaline-IS) (medium mass error 0.33 ppm).

### Sample preparation

The frozen plasma samples were thawed at room temperature prior to analysis. Aliquots of 200 μL of blank plasma (plasma free of analytes and IS) were transferred to 2 mL plastic centrifuge tubes. Fifty microliters of the analytes working solutions were spiked into the plasma samples to achieve concentrations within the ranges of the calibration curve and quality control samples (concentration of the spiking solution for the standards are demonstrated in S1 Table). Fifty microliters of the ISs intermediate standard solution, in the concentration of 1500 ng.mL^−1^ for sulfaquinoxaline and 4000 ng mL^−1^ for isoniazid, were also added to the plasma samples, in order to obtain a fixed concentration of 75 ng.mL^−1^ sulfaquinoxaline and 200 ng mL^−1^ isoniazid. The samples were placed on a vortex for 1 min. Then, 700 μL of acetonitrile (containing 0.1% formic acid) was added to the tube, and the samples were shaken again for 3 min. Samples were centrifuged at 14,000 rpm and 4°C for 10 min (Eppendorf 5810R, Hamburg, Germany), and 500 μL of supernatant was transferred to 2.0 mL glass vials and diluted to 1:2 (v/v) with ultrapure water. Then, the samples were centrifuged once more at 4,000 rpm and 4°C for 10 min, and placed into an autosampler rack for injection into the chromatographic system.

### Method validation

The method was validated for the parameters of limit of detection (LOD), lower limit of quantification (LLOQ), selectivity, calibration curve, precision, accuracy, carry-over, recovery, matrix effect, and stability according to FDA, EMA, and ANVISA guidelines for bioanalytical method validation [17–19].

#### Limit of detection (LOD) and lower limit of quantification (LLOQ)

The limits of detection and quantification were determined according to a signal-to-noise ratio of 3:1 for LOD and at least 5:1 for LLOQ. Plasma samples were spiked in triplicate with decreasing concentrations of analytes, until the lowest concentration with desired precision (RSD < 20%) was achieved.

#### Selectivity

Six blank plasma samples (free of analytes and IS) from different sources (four normal, one lipemic, and one hemolyzed plasma) were compared to a plasma sample spiked with analytes (at LLOQ concentrations) and IS.

#### Calibration curve

Calibration curves were constructed for three consecutive days using internal standards. The range of the calibration curve was based on therapeutic range and plasma concentration of antidiabetic agents found in others studies in literature [20–31]. Eight concentration levels of analytes were prepared in triplicate from serial dilutions of working solutions of NAT, PIO, and ROS. Nine concentration levels of analytes were prepared in triplicate from serial dilutions of working solutions of CHL, GBC, GCZ, GMP, MET, and VDP. The calibration curves contained a blank plasma sample (processed matrix sample without IS) and a zero sample (processed matrix sample with IS). The concentration ranges obtained for each analyte were as follows: 50–1000 ng.mL^-1^ for GCZ; 125–1000 ng.mL^-1^ for ROS; 125–2000 ng.mL^-1^ for GBC, GMP, and VDP; 250–2000 ng.mL^-1^ for PIO; 250–4000 ng.mL^-1^ for MET; 500–4000 ng.mL^-1^ for NAT; and 500–4500 ng.mL^-1^ for CHL. The IS were added to each concentration level in fixed concentrations of 75 ng.mL^-1^ sulfaquinoxaline (SPQ) and 200 ng.mL^-1^ isoniazid (ISZ). The calibration curves were built by weighted 1/x regression analysis of the peak area ratios of the analyte/IS against the analyte/IS nominal concentration. Regression parameters such as the linear equation, slope, intercept, and correlation coefficient (r) were calculated. Variation of up to 15% in the accuracy and precision at each level was allowed, except for LLOQ, for which maximum variations of 20% were permitted.

#### Accuracy and precision

To monitor the performance of the analytical method, accuracy and precision were assessed by analysis of quality control (QC) samples in five replicates at four concentration levels of the analytes, as shown in Table 1. Dilution quality control (DQC) samples were also prepared in quintuplicate, in a manner to obtain concentrations ten times higher than the medium quality controls (MQC). DQC samples were diluted 1:10 (v/v) with blank plasma in order to fit in the calibration curve. After dilution, these samples were processed and analyzed.

**Table 1:** Quality control levels used to evaluate method performance.

Analytes	LLOQ (ng/mL)	LQC (ng/mL)	MQC (ng/mL)	HQC (ng/mL)	DQC (ng/mL)
**Chlorpropamide**	500	1000	2500	3500	25000
**Glibenclamide**	125	250	1000	1500	10000
**Gliclazide**	50	125	500	750	5000
**Glimepiride**	125	250	1000	1500	10000
**Metformin**	250	500	2000	3000	20000
**Nateglinide**	500	1000	2000	3000	20000
**Pioglitazone**	250	500	1000	1500	1000
**Rosiglitazone**	125	250	500	750	5000
**Vildagliptin**	125	250	1000	1500	10000

LLOQ, lower limit of quantification; LQC, low quality control; MQC, medium quality control; HQC, high quality control; DQC, dilution quality control.

The intra-day and inter-day accuracies were evaluated over three consecutive days, by calculating the difference between the theoretical and experimental concentrations (relative error, RE%). For this purpose, five replicates of the QC samples were prepared for each analytical run, according to the concentrations shown in Table 1.

Precision was determined by the residual standard deviation (RSD%) at each QC level.

The variation of RE% and RSD% should not have exceeded 15%, except for the LLOQ, for which the deviation should not have exceeded 20%.

#### Carry-over

To evaluate the carry-over effect, a blank plasma sample was injected into the chromatographic system, followed by injection of a plasma sample spiked with analyte concentrations of the highest calibration levels. Then, the blank plasma sample was injected two more times. The acceptance criteria was that the responses of the interfering peaks in the blank sample must be less than 5% of the peak area of the ISs and less than 20% of the peak area of the compounds of interest in samples processed at the LLOQ concentration.

#### Recovery and matrix effect

Recovery was measured by comparing plasma samples spiked with analytes and ISs prior to sample clean up with standard solution samples at the same concentration, which represented 100% recovery. This assay was performed in quintuplicate at three levels: LQC, MQC, and HQC (Table 1).

The matrix effect (ME) was assessed by comparing the response obtained from plasma samples to which analytes were added post-extraction (i.e., plasma samples that were submitted to the clean-up procedure prior to addition of the analytes and IS) to standard solution samples at the same concentration. The evaluation of the ME was performed using eight replicates of QC samples at two levels, LQC and HQC (Table 1). For each level, the normalized effect of the matrix (NEM) was assumed as the response of the analyte/IS in the matrix divided by the response of the analyte/IS in the solution. Variations higher than 15% relative to the NEM calculated for all samples suggested the presence of the matrix effect.

#### Stability

The stability tests of compounds of interest and IS in plasma samples were performed under various conditions: benchtop stability (room temperature for 6 h before sample clean up), long-term stability (-40°C for 30 d before sample clean up), processed sample stability (room temperature for 8 h after sample clean up), and freeze and thaw stability (three freeze-thawed cycles at -40°C for 12 h before sample clean up). Plasma stability was evaluated by comparing the response of the analytes and the ISs obtained from the stored samples with the mean values obtained from freshly prepared samples at the same concentration levels (LQC and HQC, Table 1) in triplicate.

The stability of stock solutions (1 mg.mL^-1^ of each analyte and IS) was determined after 30 d of storage at -40°C, after 6 h at room temperature, and after 72 h at 4°C (1000 ng.mL^-1^ of each analyte and IS).

Plasma samples were considered stable when the responses were within the 15% range of the nominal value, and standard solution samples were considered stable when the deviation from the theoretical value was less than 10%.

#### Ethics statement and volunteer blood collection

The above method was applied to the plasma samples of diabetic patients and healthy volunteers. Samples of 40 diabetic patients were collected to routine analyses and were donated for this study. Sixteen non-diabetic volunteers participated in the study after signing a written consent form.

In Free and Clarified Consent Term (FCCT) were addressed the objective of the study, possible risks related to the study, the blood collection procedure and place, and informed who was the responsible for the research and gave his telephone and e-mail contact. Moreover, was explained trough FCCT that volunteers would not have any responsibility in study expenses and that the no personal information would be disclosed, and only the members of the study would have access to it. After reading and agreeing with the terms, the volunteers signed the FCCT.

Each pair of volunteers received a single dose of one of the following drugs: Diabinese^®^ (chlorpropamide 250 mg), Glibeneck^®^ (glibenclamide 5 mg), Azukon MR^®^ (gliclazide 30 mg), Betes^®^ (glimepiride 4 mg), Metformin Hydrochloride 500 mg, Starlix^®^ (nateglinide 120 mg), Piotaz^®^ (pioglitazone hydrochloride 30 mg), and Galvus^®^ (vildagliptin 50 mg). Diabinese^®^, Glibeneck^®^, Azukon MR^®^, Betes^®^, and Starlix^®^ were taken before breakfast, and Metformin Hydrochloride, Piotaz^®^, and Galvus^®^ were taken after breakfast.

Two and a half hours after the oral drug administration, blood samples (3.0 mL) were collected in tubes containing ethylenediaminetetraacetic acid as anticoagulant. All samples were immediately centrifuged at 4,000 rpm for 6.0 min at room temperature, and the plasma was separated and stored at −40°C before analysis.

Rosiglitazone was not administrated because this drug is not commercially available in Brazil.

The Ethics Committee of *Hospital de Clínicas da Universidade Federal do Paraná* (Clinics Hospital of the University of Paraná) approved this experimental protocol.

#### Volunteer plasma sample preparation

The human plasma samples were thawed at room temperature. Aliquots of 200 μL were pipetted into 2 mL polypropylene tubes and spiked with 50 μL of acetonitrile:water (70:30, v/v) and 50 μL of the IS solution to obtain a final concentration of 200 ng mL^−1^ isoniazid and 75 ng mL^−1^sulfaquinoxaline. The samples were placed on a vortex for 1 min. Then, 700 μL of acetonitrile containing 1% formic acid as additive was added, and the mixture was vortexed for 3 min and centrifuged at 14,000 rpm and 4°C for 10 min to extract the matrix compounds. The supernatants were diluted with water (1:2, v/v) and injected into the analytical system.

## Results and discussion

### Method development

The first step in method development was the optimization of spectrometry parameters by injecting CHL, GBC, GCZ, GMP, MET, NAT, PIO, ROS, VDP, and ISs (isoniazid and sulfaquinoxaline) individually into the mass spectrometer. In this phase, the ESI source parameters were adjusted to achieve the best signal intensities and signal stabilities for all analytes and ISs. Positive ionization mode was chosen because the analytes had functional groups that readily accept a proton [H^+^], such as amines, amides, and esters (Fig 1). Formic acid, ammonium formate, and combinations of the two were tested as ionization enhancers, and 0.1% formic acid was selected because it provided the highest signal intensities for all nine analytes and both ISs.

**Fig 2:**
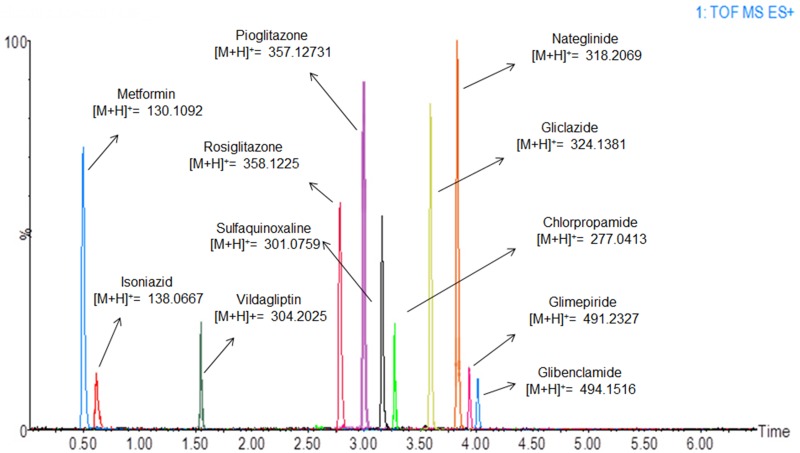
Extracted MS chromatograms of the standards in plasma.

Regarding the chromatographic method, columns with different polarities were tested (C8, C18, and cyano columns). The Acquity UPLC^®^ HSS Cyano column (100 x 2.1 mm, 1.8 μm) was chosen because it separated all the drugs used in the study with baseline resolution. When analytes with substantial differences in polarity are analyzed in a single run, a stationary phase with an intermediate polarity (such as a cyano column) is usually a better choice. For instance, in this study we looked at metformin, which is highly polar (Kow = −2.64), and glibenclamide, which is substantially nonpolar (Kow = 4.79) [32]. Different mobile phase compositions were tested using water and acetonitrile or methanol, all containing 0.1% formic acid. Acetonitrile was chosen as the organic modifier because it provided better ionization of the analytes than methanol. The gradient elution was chosen to approximate the analytes′ retention time, improve peak resolution, and reduce run time. Desolvation gas temperature and flow, cone gas flow, column temperature, and injection volume were also optimized. The chromatogram obtained under the defined conditions is shown in Fig 2, with mass error under 5 ppm.

Of all the methods found in the literature that were validated to quantify oral antidiabetic agents in human plasma [30–56], this method is the only one that encompasses nine drugs from five different classes in a very short run time, with only 4.5 min for chromatographic separation and 2 min for cleaning of the column and re-equilibration to initial conditions. The use of UPLC instead of HPLC results in a fast analysis. Because the UPLC system withstands higher pressures, it is compatible with stationary phases with a reduced particle size and length, which generates high chromatographic efficiency in a shorter run time. Only two other studies have employed UPLC systems for the quantification of oral antidiabetic agents in plasma [39, 54], but only five analytes were analyzed in each study, from just one [54] and two therapeutic classes [39].

### Sample cleanup

Protein precipitation (PP) was chosen as the extraction procedure because it is a simple, fast, and cheap technique. Furthermore, the antidiabetic agents in the study have different polarities and PP is compatible with both hydrophilic and hydrophobic compounds simultaneously. Four precipitant agents were tested: acetonitrile, acetonitrile with 0.1% formic acid, methanol, and methanol with 0.1% formic acid. The highest rates of recovery of analytes and reproducible results were obtained with acetonitrile with 0.1% formic acid.

### Method validation

#### Limit of detection (LOD) and lower limit of quantification (LLOQ)

The present method proved to be highly sensitive, as demonstrated by the low LODs of 1.25 ng.mL^-1^ for GBC, GCZ, PIO, ROS, and VDP; 2.5.ng mL^-1^ for GMP and MET; 5 ng.mL^-1^ for NAT; and 10 ng.mL^-1^ for CHL. The LLOQs were estimated to be 5.0 ng.mL^-1^ for GCZ; 12.5 ng.mL^-1^ for GBC, GMP, ROS, and VDP; 25.0 ng.mL^-1^ for MET and PIO; and 50.0 ng.mL^-1^ for CHL and NAT. The LLOQ values were established as the expected values for therapeutic monitoring studies [21–28].

#### Selectivity

**Fig 3:**
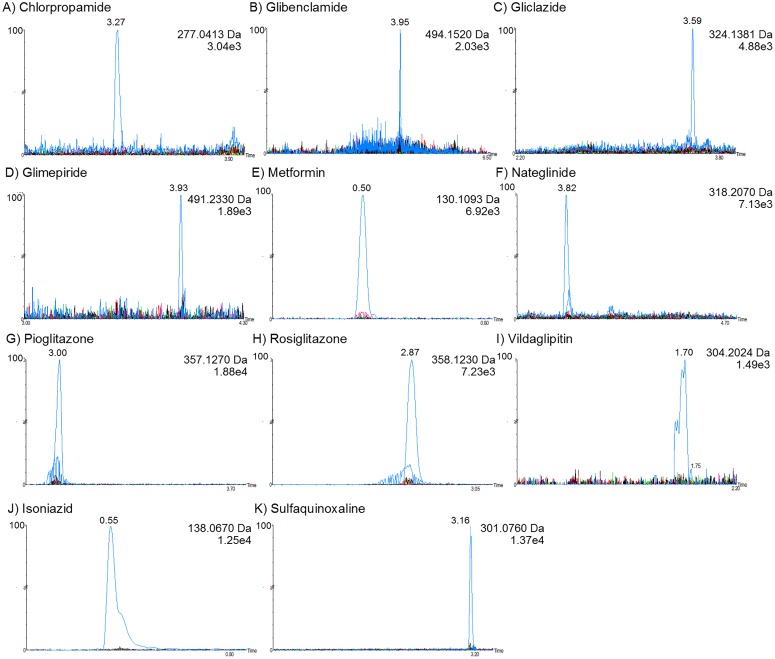
Selectivity study: Chromatograms of antidiabetic agents overlapping blank plasma chromatograms. (A) chlorpropamide; (B) glibenclamide; (C) gliclazide; (D) glimepiride; (E) metformin; (F) nateglinide; (G) pioglitazone; (H) rosiglitazone; (I) vildagliptin; (J) isoniazid (IS); and (K) sulfaquinoxaline (IS).

The method was demonstrated to be selective, as no interfering peaks appeared in the retention times of the analytes and ISs in blank, hemolyzed, or lipemic plasma (Fig 3).

#### Calibration curve

**Fig 4:**
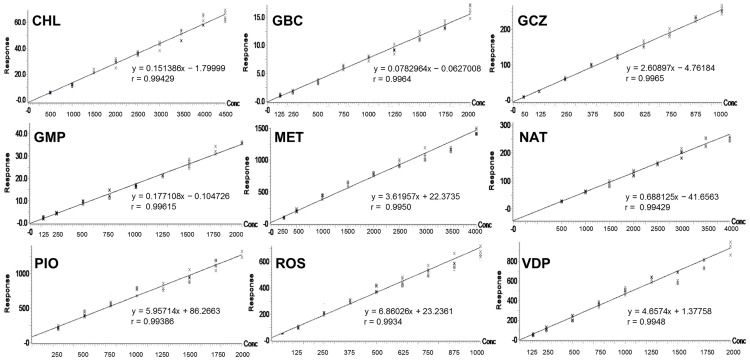
Calibration curve of analytes in plasma sample.

Calibration curves obtained in plasma were linear with the correlation coefficients (r) > 0.99. The slope, intercept, and correlation coefficients of each analyte are demonstrated in Fig 4. At all levels, RE% and RSD% deviated less than 15%. Hence, the results are in agreement with the recommendations of the guidelines.

#### Accuracy and precision

The method was accurate and precise for all analytes and ISs, with REs and RSDs of less than 15.0% for both intra- and inter-day analyses. The assays also showed that diluted samples exhibited similar accuracy and precision to the undiluted samples. The results for accuracy and precision are displayed in Table 2.

**Table 2:** Precision and accuracy of the antidiabetic agents and internal standards obtained in human plasma by the UPLC-QToF method.

Compound	Quality control level	Concentration (ng/mL^/^)	Precision Intraday (RSD%)	Accuracy Intraday (RE%)	Precision Interday (RSD%)	Accuracy Interday (RE%)
**Chlorpropamide**	LLOQ	500	6.95	3.92	6.74	3.49
	LQC	1000	8.20	0.54	8.01	-3.35
	MQC	2500	4.61	-1.26	6.13	0.82
	HQC	3500	6.87	3.50	7.40	2.90
	DQC	2500	4.74	11.11	9.24	6.19
**Glibenclamide**	LLOQ	125	8.03	5.76	11.02	4.96
	LQC	250	6.10	-1.84	7.61	-2.24
	MQC	1000	4.63	-0.02	8.36	-6.97
	HQC	1500	5.83	-1.21	9.08	1.62
	DQC	10000	3.53	11.82	6.43	10.62
**Gliclazide**	LLOQ	50	7.64	8.80	7.01	10.02
	LQC	125	7.92	3.36	4.77	-6.66
	MQC	500	2.81	-8.52	4.46	-5.29
	HQC	750	2.55	3.62	3.83	1.88
	DQC	5000	4.35	6.10	5.66	3.29
**Glimepiride**	LLOQ	125	-3.74	8.80	11.31	2.82
	LQC	250	-5.69	3.04	7.16	0.42
	MQC	1000	5.97	-1.88	6.82	-6.98
	HQC	1500	-1.48	7.71	5.76	3.09
	DQC	10000	5.81	1.33	7.40	-4.10
**Metformin**	LLOQ	250	10.41	-2.56	11.45	-11.44
	LQC	500	8.86	-1.96	10.71	4.73
	MQC	2000	5.02	10.6	5.19	7.31
	HQC	3000	6.25	-0.01	5.75	-1.15
	DQC	20000	8.92	-4.98	9.17	-5.08
**Nateglinide**	LLOQ	500	1.84	-2.32	4.97	2.42
	LQC	1000	1.82	-0.24	5.92	-2.90
	MQC	2000	2.52	1.37	6.16	-3.13
	HQC	3000	2.39	-1.58	5.01	2.12
	DQC	20000	4.15	-5.65	7.82	-6.21
**Pioglitazone**	LLOQ	250	4.09	-11.11	8.19	-10.57
	LQC	500	5.76	8.96	7.01	6.80
	MQC	1000	4.78	-0.30	8.62	6.68
	HQC	1500	5.37	-3.30	6.63	-2.98
	DQC	10000	5.10	5.75	7.14	3.50
**Rosiglitazone**	LLOQ	125	10.25	-10.82	8.86	-11.50
	LQC	250	9.34	8.40	5.59	7.80
	MQC	500	6.13	8.08	5.97	5.91
	HQC	750	9.05	-3.48	6.41	-2.02
	DQC	5000	3.72	0.63	4.65	2.26
**Vildagliptin**	LLOQ	125	6.49	-2.72	11.58	-2.45
	LQC	250	8.50	-1.12	8.02	0.14
	MQC	1000	10.53	2.02	9.09	1.27
	HQC	1500	5.78	4.64	7.83	1.09
	DQC	10000	11.90	-4.50	11.52	-3.32
**Isoniazid (IS)**	-	200	7.32	-0.02	8.24	-0.12
**Sulfaquinoxaline (IS)**	-	75	11.11	6.20	11.10	1.69

LLOQ, lower limit of quantification; LQC, low quality control; MQC, medium quality control; HQC, high quality control; DQC, dilution quality control IS, internal standard; RE%, relative error; RSD%, relative standard deviation. Intraday analysis, n = 5; interday analysis, n = 15.

#### Carry-over

The responses of interfering peaks observed at the retention times of the analytes and ISs in the blank injected right after the highest calibration level were less than 20% and 5%, respectively. Therefore, no carry-over effect was observed between injections.

#### Recovery and matrix effect

The individual extraction recoveries and matrix effect data are shown in Table 3. The recovery rates ranged from 61.30 to 86.54%, with RSDs of less than 10%. Therefore, the extraction method using acetonitrile containing 0.1% formic acid can be considered effective and robust for extracting oral antidiabetic agents from human plasma.

**Table 3:** Recovery and matrix effect of the antidiabetic agents and internal standards.

Compound	Quality control level	Concentration (ng/mL)	Recovery (%)	RSD (%)	Matrix effect (NEM%)
**Chlorpropamide**	LQC	1000	83.32	6.13	11.28
	MQC	2500	86.54	9.32	-
	HQC	3500	84.54	7.48	11.74
**Glibenclamide**	LQC	250	75.33	5.35	11.24
	MQC	1000	75.05	6.05	-
	HQC	1500	80.43	7.43	9.60
**Gliclazide**	LQC	125	84.52	4.32	11.71
	MQC	500	80.55	5.96	-
	HQC	750	80.37	5.92	7.69
**Glimepiride**	LQC	250	78.43	7.64	8.86
	MQC	1000	82.96	2.17	-
	HQC	1500	80.35	6.05	10.87
**Metformin**	LQC	500	61.60	8.82	7.53
	MQC	2000	62.56	9.54	-
	HQC	3000	67.37	9.03	10.08
**Nateglinide**	LQC	1000	75.05	5.76	12.78
	MQC	2000	71.03	8.51	-
	HQC	3000	76.31	7.33	4.80
**Pioglitazone**	LQC	500	79.94	1.40	5.09
	MQC	1000	75.85	4.23	-
	HQC	1500	76.88	3.93	4.47
**Rosiglitazone**	LQC	250	80.95	2.57	9.09
	MQC	500	83.43	3.66	-
	HQC	750	83.91	3.03	4.04
**Vildagliptin**	LQC	250	73.03	8.37	13.41
	MQC	1000	76.65	5.37	-
	HQC	1500	73.32	7.03	12.83
**Isoniazid (IS)**	-	200	77.43	3.94	-
**Sulfaquinoxaline (IS)**	-	75	84.54	2.89	-

LQC, low quality control; MQC, medium quality control; HQC, high quality control; IS, internal standard; RSD%, relative standard deviation; NEM, normalized effect of matrix.

The normalized effect of the matrix exhibited coefficient variation below 15%, demonstrating that other components of the sample do not interfere with the analysis.

#### Stability

The stability tests proved that the analytes and ISs were stable in the biological matrix for 6 h at room temperature (bench-top stability), for 30 d at -40°C (long-term stability), for 8 h in the sample manager at room temperature (processed sample stability), and after three freeze–thaw cycles (12 h-long cycles at -40°C). Moreover, the assays showed that the working standard solutions of the analytes and ISs were stable for 6 h at room temperature (25°C), and for 72 h at 4°C. The compounds were stable in the stock solutions for 28 d at -40°C.

### Method application

The developed UPLC-QToF-MS method was successfully applied to real plasma samples from diabetic patients from the Clinics Hospital of Paraná, and to samples from healthy volunteers who received an oral dose of one of the drugs in the study. The linear equations and correlation coefficients obtained during sample analyses are demonstrated in S1 Fig. Moreover, all QC levels exhibited deviations of less than 15.0%. The concentrations of drugs in plasma samples obtained from patients treated with glibenclamide, gliclazide, glimepiride, metformin, and vildagliptin are summarized in Table 4. These values are in agreement with the therapeutic range and plasma concentrations observed in previous studies [21–28, 31]. The mean plasma concentrations of the analytes observed in volunteers’ samples are summarized in Table 5. These values are in agreement with other results reported in the literature [20, 29, 30].

**Table 4:** Amounts of glibenclamide, gliclazide, glimepiride, metformin, and vildagliptin in plasma of diabetic patients (n = 40).

	GBC ng/mL (n = 11)	GCZ ng/mL (n = 2)	GMP ng/mL (n = 6)	MET ng/mL (n = 19)	VDP ng/mL (n-2)
**Mean ± SD**	128.2 ± 72.9	1,515.5 ± 10.6	315.7 ± 80.1	1,107.0 ± 262.8	323.5 ± 41.7

GBC, glibenclamide; GCZ, gliclazide; GMP, glimepiride; MET, metformin; VDP, vildagliptin.

**Table 5:** Amounts of chlorpropamide, glibenclamide, gliclazide, glimepiride, metformin, nateglinide, pioglitazone, rosiglitazone, and vildagliptin in plasma of volunteers (n = 16).

	CHL ng/mL (n-2)	GBC ng/mL (n-2)	GCZ ng/mL (n-2)	GMP ng/mL (n-2)	MET ng/mL (n-2)	NAT ng/mL (n-2)	PIO ng/mL (n-2)	VDP ng/mL (n-2)
**Mean ± SD**	25,590 ± 2,150	219 ± 48	1,364 ± 35	170 ± 27	1,007 ± 148	4,184 ± 297	1,416 ± 192	403 ± 87

CHL, chlorpropamide; GBC, glibenclamide; GCZ, gliclazide; GMP, glimepiride; MET, metformin; NAT, nateglinide; PIO, pioglitazone; ROS, rosiglitazone; VDP, vildagliptin.

The results indicate the high robustness and selectivity of the method, as well as its capability to quantify analytes in samples from patients with different metabolic profiles and comorbidities collected at different times. These characteristics reveal that the method can be used for routine analysis for therapeutic monitoring. Therapeutic monitoring allows for individualized drug dosages and personalized medical care. It provides the ideal drug therapy approach for diabetes by considering metabolic variations among patients, as diabetes is a complex pathology that involves many organs and systems. Furthermore, it is known that genetic factors may significantly interfere with plasma distribution of sulfonylurea [57] and metformin [58, 59]. Reitman and Schadt (2007) [60] reported the growing need for personalized medicine for diabetic patients because of large metabolic and genetic differences among individuals.

In addition, the results showed significant differences among the plasmatic concentrations of volunteers who received the drugs. Although the volunteers were all healthy, received the antidiabetic agents at the same time, and had blood collected in pairs at the same time, metabolic variations caused differences in plasma concentrations of up to 35% after drug administration, as can be seen in Table 5. Through these analyses, we report a real need for therapeutic monitoring of oral antidiabetic agents. Significant variations suggest that despite similar doses, metabolic rates of the drugs may vary among patients. With the aid of plasma quantification, health professionals will have a powerful tool for the establishment of more effective pharmacological treatments with a reduced likelihood of adverse events related to therapy.

Therefore, the present method was proven to be capable of quantifying different oral antidiabetic agents in human plasma from single and multiple doses, and to be suitable for pharmacokinetic, bioavailability, bioequivalence, and therapeutic monitoring studies.

## Conclusions

A method using a UPLC-QToF-MS analyzer was developed and validated for simultaneous quantification of chlorpropamide, glibenclamide, gliclazide, glimepiride, metformin, nateglinide, pioglitazone, rosiglitazone, and vildagliptin in human plasma; these drugs represent the main five classes of oral antidiabetic agents available on the market. The new method was proven to be selective, sensitive, linear, accurate, precise, and free of residual and matrix effects. A simple, fast, and reproducible sample preparation method using protein precipitation was also developed. The method was successfully applied to patient samples, and the presence of different antidiabetic agents could be determined. The new method can be considered suitable for pharmacokinetic, bioavailability, bioequivalence, and therapeutic monitoring studies of oral antidiabetic agents.

## Supporting Information

S1 Fig Calibration curve of analytes in plasma sample during method application. (TIF)

S1 Table Concentrations of the spiking solutions for the standards used to prepare each calibration level and quality control levels. CHL, chlorpropamide; GBC, glibenclamide; GCZ, gliclazide; GMP, glimepiride; MET, metformin; NAT, nateglinide; PIO, pioglitazone; ROS, rosiglitazone; VDP, vildagliptin.
(DOC)
