# Supplementary figures and images for: Simultaneous Quantification of Antidiabetic Agents in Human Plasma by a UPLC–QToF-MS Method
Source: PLoS One. 2016 Dec 8;11(12):e0167107. doi: 10.1371/journal.pone.0167107 (PMC5145167; doi:10.1371/journal.pone.0167107)

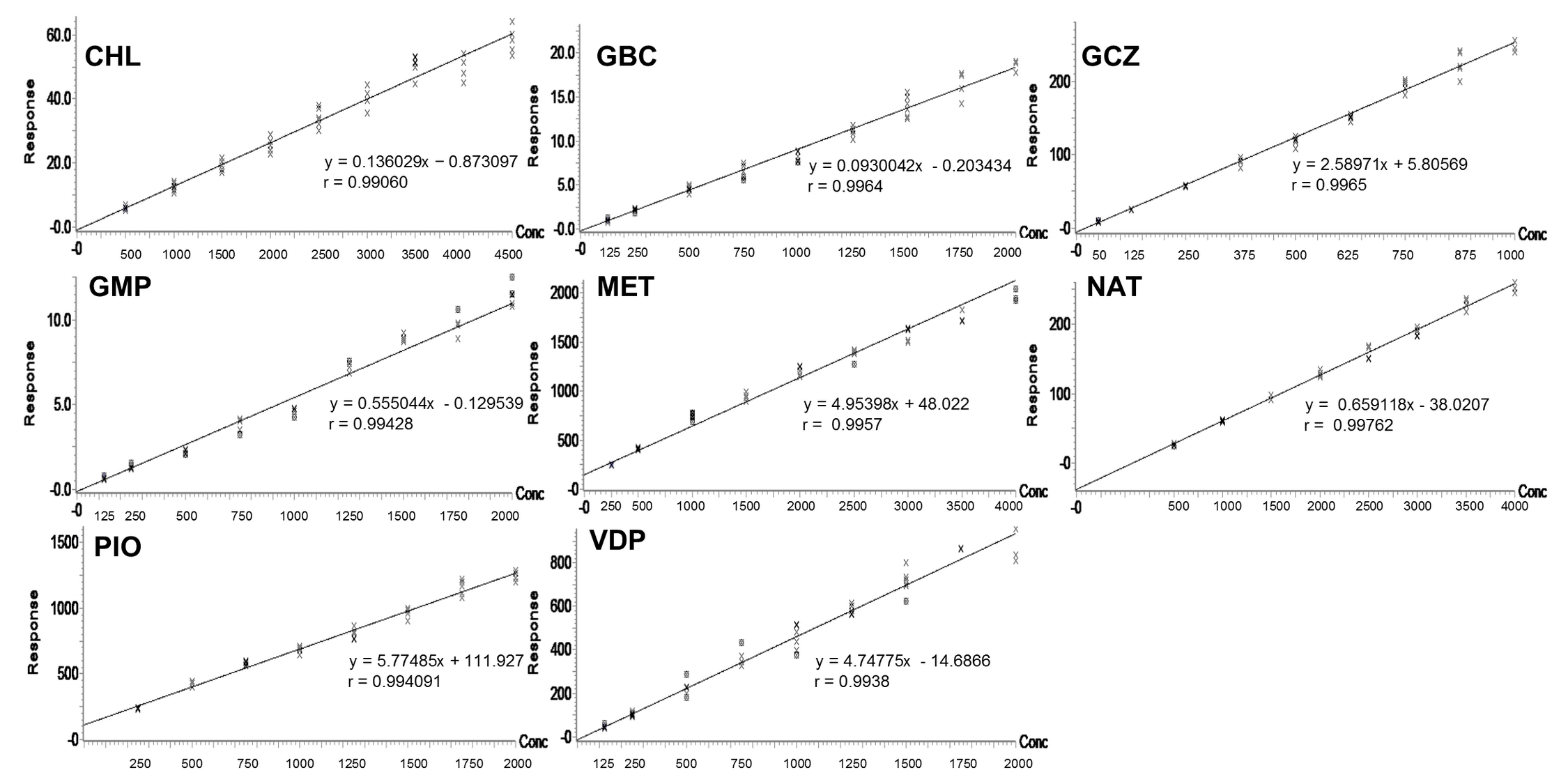

Supplement: S1 Fig — (TIF) [file pone.0167107.s001.tif]
