# Supplementary material for: Simultaneous Quantification of Antidiabetic Agents in Human Plasma by a UPLC–QToF-MS Method
Source: PLoS One. 2016 Dec 8;11(12):e0167107. doi: 10.1371/journal.pone.0167107 (PMC5145167; doi:10.1371/journal.pone.0167107)
Supplement: S1 Table — CHL, chlorpropamide; GBC, glibenclamide; GCZ, gliclazide; GMP, glimepiride; MET, metformin; NAT, nateglinide; PIO, pioglitazone; ROS, rosiglitazone; VDP, vildagliptin. (DOC) [file pone.0167107.s002.doc]

**S1 Table. Concentrations of the spiking solutions for the standards used to prepare each calibration level and quality control levels.**

| **Calibration Level** | **CHL (µg.mL−1­­)** | **GBC (µg.mL−1­­)** | **GCZ (µg.mL−1­­)** | **GMP (µg.mL−1­­)** | **MET (µg.mL−1­­)** | **NAT (µg.mL−1­­)** | **PIO (µg.mL−1­­)** | **ROS (µg.mL−1­­)** | **VDP (µg.mL−1­­)** |
| --- | --- | --- | --- | --- | --- | --- | --- | --- | --- |
| **1** | 2.0 | 0.5 | 0.2 | 0.5 | 1.0 | 2.0 | 1.0 | 0.5 | 0.5 |
| **2** | 4.0 | 1.0 | 0.5 | 1.0 | 2.0 | 4.0 | 2.0 | 1.0 | 1.0 |
| **3** | 6.0 | 2.0 | 1.0 | 2.0 | 4.0 | 6.0 | 3.0 | 1.5 | 2.0 |
| **4** | 8.0 | 3.0 | 1.5 | 3.0 | 6.0 | 8.0 | 4.0 | 2.0 | 3.0 |
| **5** | 10.0 | 4.0 | 2.0 | 4.0 | 8.0 | 10.0 | 5.0 | 2.5 | 4.0 |
| **6** | 12.0 | 5.0 | 2.5 | 5.0 | 10.0 | 12.0 | 6.0 | 3.0 | 5.0 |
| **7** | 14.0 | 6.0 | 3.0 | 6.0 | 12.0 | 14.0 | 7.0 | 3.5 | 6.0 |
| **8** | 16.0 | 7.0 | 3.5 | 7.0 | 14.0 | 16.0 | 8.0 | 4.0 | 7.0 |
| **9** | 18.0 | 8.0 | 4.0 | 8.0 | 16.0 | - | - | - | 8.0 |

CHL, chlorpropamide; GBC, glibenclamide; GCZ, gliclazide; GMP, glimepiride; MET, metformin; NAT, nateglinide; PIO, pioglitazone; ROS, rosiglitazone; VDP, vildagliptin.
